# Blocking siglec-10^hi^ tumor-associated macrophages improves anti-tumor immunity and enhances immunotherapy for hepatocellular carcinoma

**DOI:** 10.1186/s40164-021-00230-5

**Published:** 2021-06-10

**Authors:** Nan Xiao, Xiaodong Zhu, Kangshuai Li, Yifan Chen, Xuefeng Liu, Bin Xu, Ming Lei, Jiejie Xu, Hui-Chuan Sun

**Affiliations:** 1 Department of Liver Surgery and Transplantation, Liver Cancer Institute and Zhongshan Hospital, Fudan University, Shanghai, 200032 China; 2 Key Laboratory of Carcinogenesis and Cancer Invasion (Fudan University), Ministry of Education, Shanghai, China; 3 Department of Hepatobiliary Surgery, Qilu Hospital of Shandong University, Jinan, 250012 China; 4 Department of Immunology, School of Basic Medical Sciences, Fudan University, Shanghai, China; 5 Department of Biochemistry and Molecular Biology, School of Basic Medical Sciences, Fudan University, Shanghai, 200032 China

**Keywords:** Macrophages, Tumor microenvironment, Hepatocellular carcinoma, Immunotherapy, Immune evasion

## Abstract

**Background:**

Tumor-associated macrophages (TAMs) promote key processes in the modulation of tumor microenvironment (TME). However, the clinical significance of heterogeneous subpopulations of TAMs in hepatocellular carcinoma (HCC) remains unknown.

**Methods:**

HCC tissues from Zhongshan Hospital and data from The Cancer Genome Atlas were obtained and analyzed. Immunohistochemistry and flow cytometry were performed to detect the characteristics of sialic acid-binding immunoglobulin-like lectin 10^high^ (Siglec-10^hi^) TAMs and explore their impact on the TME of HCC. The effect of Siglec-10 blockade was evaluated in vitro based on fresh human tumor tissues.

**Results:**

Our data revealed that Siglec-10 was abundant in a large proportion of HCC specimens and prominently distributed on macrophages. Kaplan–Meier curves and Cox regression analysis showed that intratumoral Siglec-10^+^ cell enrichment was associated with unfavorable prognosis in patients with HCC. Notably, multiple anti-inflammatory cytokines and inhibitory receptors were enriched in Siglec-10^hi^ TAMs. RNA sequencing data also revealed that numerous M2-like signaling pathways were significantly upregulated in Siglec-10^hi^ TAMs. High infiltration of Siglec-10^hi^ TAMs was associated with impaired CD8^+^ T cell function in HCC. Of note, blocking Siglec-10 with the competitive binding antibody Siglec-10 Fc led to decreased expression of immunosuppressive molecules and increased the cytotoxic effects of CD8^+^ T cells against HCC cells. Moreover, blocking Siglec-10 promoted the anti-tumor efficacy of the programmed cell death protein 1 (PD-1) inhibitor pembrolizumab.

**Conclusions:**

Siglec-10^hi^ TAMs are associated with immune suppression in the TME, and indicate poor prognosis in patients with HCC. Targeting Siglec-10^hi^ TAMs may serve as a promising immunotherapy approach for HCC.

**Supplementary Information:**

The online version contains supplementary material available at 10.1186/s40164-021-00230-5.

## Introduction

Hepatocellular carcinoma (HCC), the most common primary liver cancer, is one of the top causes of cancer-related death worldwide [1, 2]. The current curative treatments for HCC, including surgical resection, liver transplantation, and radiofrequency ablation, are limited to patients with early-stage disease [3]. However, most patients with HCC are diagnosed in the advanced stages, and therefore have poor prognosis [4].

Immunotherapy targeting the tumor microenvironment (TME) has revolutionized tumor treatment. The results of the CheckMate 040 and KEYNOTE-224 studies were extraordinary milestones in the battle against HCC. However, the response rate to monoclonal anti-programmed cell death protein 1 (PD-1) antibody has been disappointing due to the high heterogeneity of immune evasion mechanisms [5]. Thus, it is urgent to identify novel immune modulators to restore anti-tumor immunity in HCC.

Tumor-associated macrophages (TAMs) are the most abundant immune cells within the TME, and promote vital processes in tumor progression [6]. Increased TAM infiltration is associated with poor patient outcomes in HCC [7, 8]. TAMs can generally be classified into pro-inflammatory (M1) or anti-inflammatory (M2) phenotype depending on diverse environmental stimulus. M1 TAMs act as prominent antigen-presenting cells and play critical roles in anti-tumor activity, whereas M2 TAMs are featured by higher production of anti-inflammatory cytokines and active metabolic pathways, which suppress the adaptive immune response [9]. Actually, M1 and M2 polarization are two extremes of a spectrum of activation states. TAMs often exhibit a mixed M1/M2 phenotype, with their exact point on the scale based on the specific blend of cytokines present in the TME [9]. Targeting TAMs to remodel the TME is a hotspot in tumor immunotherapy. Blocking anti-inflammatory receptors on specific macrophage subsets can reactivate anti-tumor immunity [10–12].

Sialic acid-binding immunoglobulin-like lectin 10 (Siglec-10) is widely expressed on subsets of human leukocytes, and plays an important part in distinguishing between self and non-self in the immune system [13]. As an immune checkpoint, Siglec-10 is closely involved in the regulation of macrophage phagocytosis [14], B-cell tolerance [15], impaired T-cell activation [16], and pathogen immune evasion [17]. Of note, multiple studies have shown the clear distribution of Siglec-10 on macrophages [13, 14]. Thus, we hypothesized that Siglec-10^hi^ macrophages may promote immune evasion in HCC.

In this study, we found that Siglec-10, which was associated with poor overall survival (OS) and recurrence-free survival (RFS) in patients with HCC, was predominately expressed on macrophages. Furthermore, we demonstrated that intratumoral Siglec-10^hi^ TAM infiltration was positively associated with CD8^+^ T cell inactivation. Notably, inhibition of Siglec-10 restrained the secretion of anti-inflammatory cytokines from macrophages and suppressed the expression of inhibitory receptors on CD8^+^ T cells, resulting in restored cytotoxic activities against tumor cells. Our study identifies targeting Siglec-10^hi^ TAMs as a promising therapeutic option for HCC.

## Materials and methods

### Patients and follow-up

We retrospectively evaluated 246 patients with HCC who underwent hepatectomy from 2008 to 2010. The exclusion criteria were: pathological diagnosis other than HCC or combined with other pathological types, distant metastatic disease, or unavailability of tumor tissues or follow-up information. None of the patients enrolled in this study had received neoadjuvant therapy before surgery. Two patients with mixed HCC/intrahepatic cholangiocarcinoma (ICC) and five patients with unavailable follow-up data were excluded. Four specimens were lost on tissue microarray (TMA) when immunohistochemistry (IHC) was performed. Finally, 235 eligible HCC patients were randomly divided into discovery set (n = 115) and validation set (n = 120). Patients’ clinical characteristics are shown in Additional file 1: Table S1. A detailed flow chart of patient selection is shown in Additional file 1: Fig. S1.

The clinical tumor stage was determined according to the American Joint Committee on Cancer/International Union Against Cancer (AJCC/UICC) Tumor-Node-Metastasis (TNM) staging system 8th edition. All follow-up data were collected from the date of surgery to December 2018. OS was defined as the time period between surgery and death or last follow-up. RFS was defined as the time period between surgery and the first documented recurrence or death, whichever occurred first. This study was approved by the Clinical Research Ethics Committee of Zhongshan Hospital, Fudan University (Shanghai, China). An informed consent form was signed by every patient.

### IHC and immunofluorescence

IHC and immunofluorescence (IF) staining were performed on TMAs constructed with formalin-fixed and paraffin-embedded HCC specimens. IHC staining was performed according to a previously described protocol [11]. In brief, TMAs were incubated with primary antibodies overnight at 4 °C. The staining was carried out with DAB reagent. For IF staining, the sections were incubated with two primary antibodies at 4 °C overnight, followed by incubation with FITC- and TRITC-conjugated secondary antibodies at 37 °C for 2 h. Finally, the slides were mounted with Antifade Mounting Solution containing DAPI. The details of the antibodies are listed in Additional file 1: Table S2. For negative controls, primary antibody was omitted. All stained tissues were evaluated under the Leica DM6000 B microscope (Leica Microsystems, Wetzlar, Germany) independently by two pathologists who were blinded to the patients’ characteristics.

### Fresh samples and flow cytometry

Seventy-seven fresh human specimens were collected from HCC patients, who underwent liver resection at Zhongshan Hospital. Single-cell suspension was prepared as previously described [18]. After removing blood and necrotic tissues, samples were digested in RPMI Medium 1640 with 1 mg/mL Collagenase (Sigma Aldrich) and 0.1 mg/mL DNase I (Roche) for 30 min at 37 °C. Then, the tumor suspension was filtered through a 70 mm cell strainer (BD Falcon) and washed with Stain Buffer (BD Biosciences). For intracellular cytokine detection, cells were stimulated for 4 h with phorbol myristate acetate (50 ng/mL) and ionomycin (1 µg/mL) in the presence of BD GolgiStop Protein Transport Inhibitor (1:1000). After red blood cells were removed with Lysing Buffer (BD Biosciences), Fixable Viability Dye(eBioscience)was applied to label dead cells in the samples. Then, samples were incubated with Human Fc receptor blocking reagent (BD Biosciences) and stained with the indicated fluorochrome-conjugated antibodies against surface markers for 30 min at 4 °C in dark. For staining with antibodies against intracellular proteins or transcription factors, surface-stained cells were treated with the Fixation/Permeabilization Solution Kit or Transcription Factor Fixation/Permeabilization Buffer (BD Biosciences) respectively according to the manufacturer’s instructions, and then incubated with the indicated fluorochrome-conjugated antibodies for 40 min at 4 °C in dark. Flow cytometry analysis was performed with the BD FACS Celesta flow cytometer and the data were analyzed using FlowJo 10.0 software (Tree Star). All flow cytometry antibodies and reagents are summarized in Additional file 1: Table S2.

### RNA sequencing

Siglec-10^hi^ TAMs (CD45^+^ CD14^+^ Siglec-10^hi^) and Siglec-10^lo^ TAMs (CD45^+^ CD14^+^ Siglec-10^lo^) were freshly isolated from three HCC specimens using the MoFlo XDP Cell Sorter (Beckman Coulter, Sykesville, MD, USA) and directly lysed in lysis buffer. The cDNA libraries were constructed using the TruSeq Stranded mRNA LT Sample Prep Kit (Illumina, San Diego, CA, USA) according to the manufacturer’s instructions. Then the libraries were sequenced on the Illumina HiSeq X Ten platform. About 6G raw reads for each sample of fastq format were processed using Trimmomatic [19] and low-quality reads were removed to obtain clean reads.

### Sequence analysis

The clean reads were aligned to the human genome (GRCh38) using HISAT2 [20]. The FPKM [21] of each gene was calculated using Cufflinks [22], and the read counts of each gene were obtained by HTSeq-count [23]. Differential expression analysis was performed using R software (version 3.6.0) and edgeR package (version 3.26.8) [24]. P < 0.05 and fold change > 2 or fold change < 0.5 was set as the threshold for significantly differential expression. Hierarchical cluster analysis of differentially expressed genes (DEGs) was performed to demonstrate the expression pattern of genes. Gene Ontology (GO) enrichment and Kyoto Encyclopedia of Genes and Genomes (KEGG) pathway analysis of DEGs between Siglec-10^hi^ TAMs and Siglec-10^lo^ TAMs were performed respectively using R software based on the hypergeometric distribution**.** Gene set enrichment analysis (GSEA) was performed by the Molecular Signature Database for gene functional annotation.

### Bioinformatics analysis

The Cancer Genome Atlas Liver Hepatocellular Carcinoma (TCGA-LIHC) mRNA and clinical data, including RNA sequencing (RNA-seq) and clinicopathological data for 372 tumors, were downloaded from https://www.cbioportal.org on July 15, 2020. Six cases were excluded due to lack of RNA-seq data. Finally, data from 366 patients were enrolled in this study. The median value of Siglec-10 expression was set as the cut-off value.

### In vitro neutralizing assay

In vitro intervention studies were performed according to previously described methods [25]. Dissociated from fresh human HCC tissues, single-cell suspension containing tumor cells, immune cells, and other cells were co-cultured and randomly divided into four groups (isotype control, Siglec-10 Fc, pembrolizumab, Siglec-10 Fc plus pembrolizumab). The usage of recombinant human Siglec-10 Fc chimera was according to previous research [26, 27].

The antibodies used in this experiment were IgG1 isotype control (10 μg/mL, ab206198; Abcam, Cambridge, UK), recombinant human Siglec-10 Fc chimera (5 μg/mL; R&D Systems), and pembrolizumab (5 µg/mL; Selleck Chemicals, Houston, TX, USA). After overnight culture in RPMI 1640 medium containing 10% fetal bovine serum and corresponding antibodies, cells were subjected to flow cytometry analysis to examine the apoptosis of tumor cells by using the FITC Annexin-V Apoptosis Detection Kit I (BD Biosciences) or corresponding fluorescence-activated cell sorting antibodies.

### Statistical analysis

Results are expressed as the mean ± standard deviation. Categorical variables were reported as numbers and percentages, and were analyzed by the Pearson’s chi-squared test or Fisher’s exact test. Student’s *t*-test or one-way analysis of variance was used for continuous variables. Spearman’s correlation was employed to evaluate the correlation between different variables. Kaplan–Meier method was used to determine OS and RFS. Cox proportional hazards regression model was used for multivariate analysis with hazard ratios (HRs) and 95% confidence intervals (CIs). To obtain the best prognostic efficacy, X-Tile Software (version 3.6.1; Yale University, New Haven, CT, USA) was used as previously described [28]. The cut-off values of Siglec-10 as prognostic biomarkers were defined according to the OS and RFS data of the discovery set, and then applied to the validation set. In flow cytometry analysis, the median value of intratumoral Siglec-10^hi^ TAM infiltration(Siglec-10^hi^ CD68^+^ cells / CD68^+^ cells)were defined as cut-off value. Calculations were done with SPSS 22.0 (IBM, Armonk, NY, USA). Graphical illustrations were carried out with GraphPad Prism v7 (GraphPad Software, Inc., La Jolla, CA, USA). A two-tailed P value less than 0.05 indicated statistical significance.

## Results

### Intratumoral Siglec-10^+^ cell enrichment associates with poor prognosis in patients with HCC

**Fig. 1:**
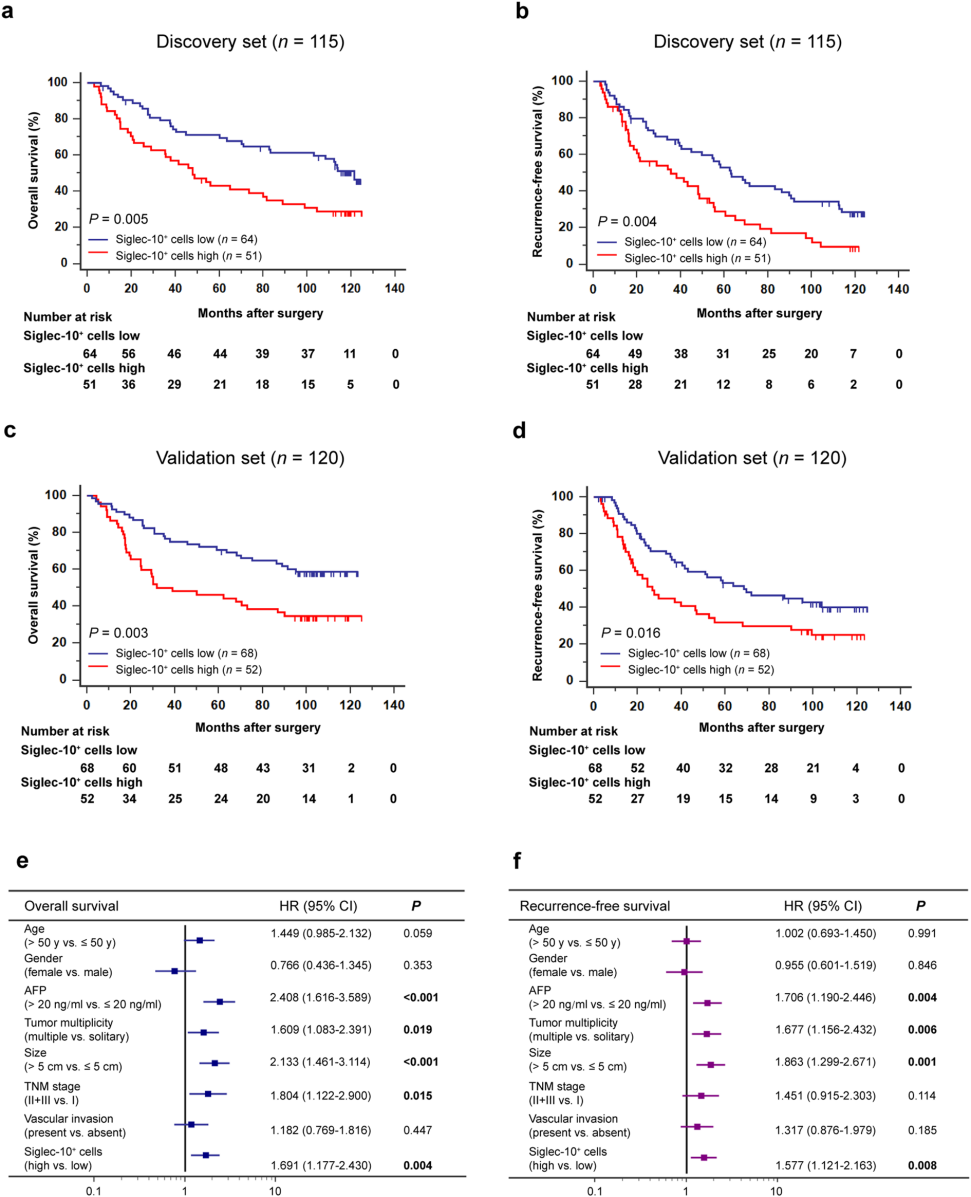
The prognostic value of intratumoral Siglec-10^hi^ cell infiltration in patients with HCC. **a**,** b** OS (**a**) and RFS (**b**) curves of patients with low and high intratumoral Siglec-10^hi^ cells infiltration in the discovery set (*n* = 115). **c**, **d** OS (**c**) and RFS (**d**) curves of patients with low and high intratumoral Siglec-10^hi^ cell infiltration in the validation set (*n* = 120). **e**, **f** Multivariate Cox proportional hazards regression analysis of OS (**e**) and RFS (**f**) for clinicopathological parameters in the total cohort including the discovery set and validation set (*n* = 235). Intratumoral Siglec-10^+^ cells were detected by IHC on TMAs

To investigate the clinical significance of intratumoral Siglec-10^+^ cells in HCC, Kaplan–Meier curves were used to compare the prognosis of patients stratified by intratumoral Siglec-10^+^ cell infiltration. Patients with low Siglec-10^+^ cell infiltration had better OS (*P* = 0.005 and 0.003, respectively; Fig. 1a, c) and RFS (*P* = 0.004 and 0.016, respectively; Fig. 1b, d) in both the discovery set and validation set. Multivariate analysis revealed that intratumoral Siglec-10^+^ cell infiltration was an independent prognostic factor of unfavorable OS and RFS (*P* = 0.004 and 0.008, HR = 1.691 and 1.577, respectively; Fig. 1e, f). These findings suggest that intratumoral Siglec-10^+^ cells may lead to poor prognosis in patients with HCC.

### Siglec-10 is enriched in HCC and mainly distributed on TAMs

**Fig. 2:**
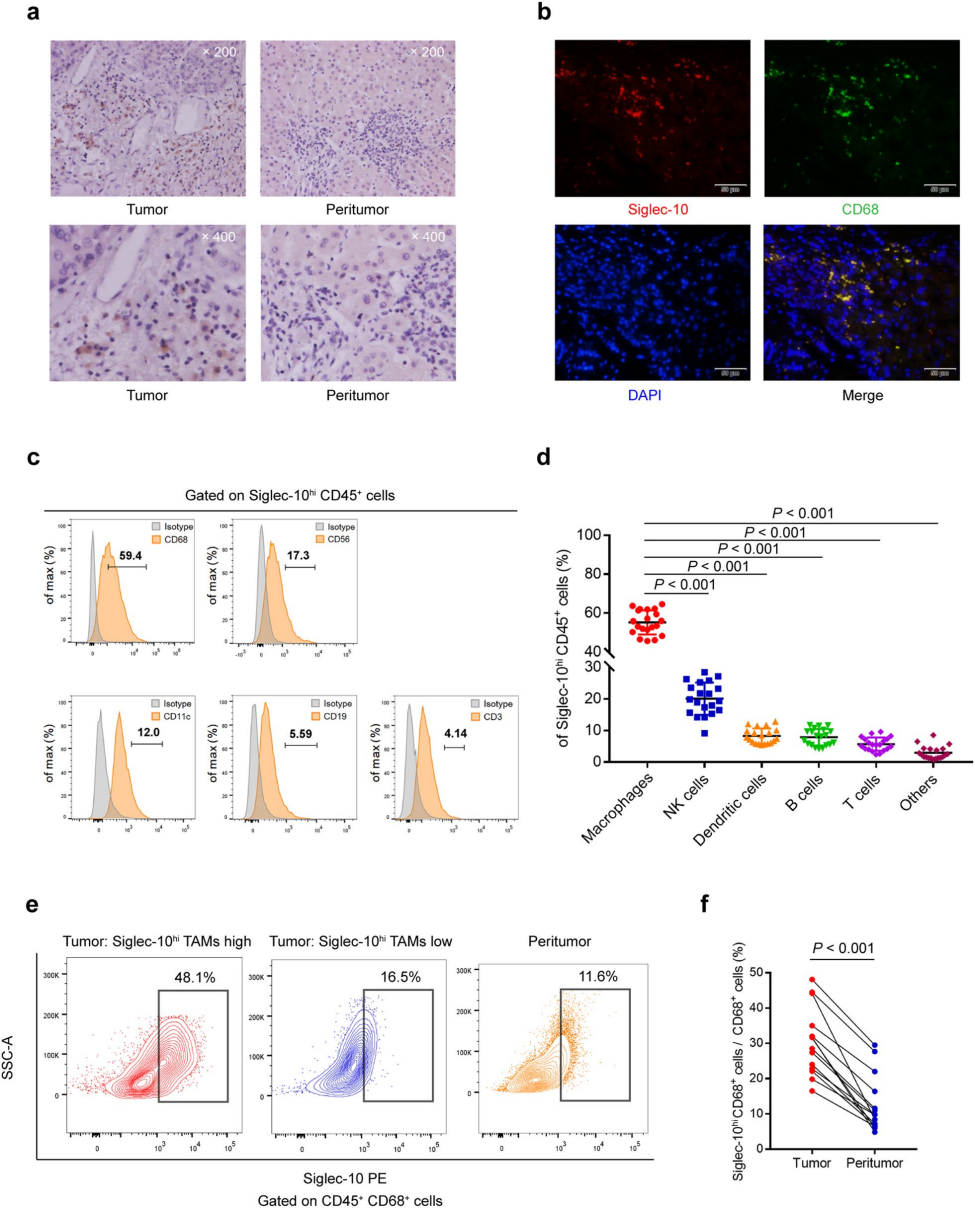
Siglec-10^hi^ cells are enriched in HCC and prominently distributed on CD68^+^ TAMs**. a** Representative images showed Siglec-10^hi^ cells in tumor and peritumor tissues. **b** HCC tissues were co-labeled with Siglec-10 (red) and macrophage marker CD68 (green). Nuclei were counterstained blue with DAPI. The lower right panel was merged by the aforementioned three images. **c**, **d** Representative flow cytometry figures (**c**) and cumulative results (**d**) showing subpopulations gated on Siglec-10^hi^ CD45^+^ leucocytes from fresh human HCC samples (*n* = 20). Bars represent the mean ± standard deviation. Data were analyzed using the Student’s *t*-test. **e**, **f** Representative images (**e**) and cumulative results (**f**) of flow cytometry showed infiltration of Siglec-10^hi^ CD68^+^ cells in total CD45^+^ CD68^+^ cells in tumor and peritumor tissues (*n* = 14). Data were analyzed using the Student’s *t*-test

IHC of HCC TMAs showed that Siglec-10^+^ cells were abundant in tumor tissues compared with peritumor tissues (Fig. 2a). IF analysis of human HCC tissues revealed that most Siglec-10 was co-localized with macrophage marker CD68 (Fig. 2b). To detect the specific distribution of Siglec-10 in the TME, the co-localization of Siglec-10 with different immune cell markers was examined. Flow cytometry showed that Siglec-10 was predominantly expressed by macrophages (Fig. 2c, d), and that HCC specimens exhibited higher levels of Siglec-10^hi^ TAMs compared with peritumor tissues (Fig. 2e, f).

### Intratumoral Siglec-10^hi^ TAMs are associated with the pro-tumor immune contexture of HCC

**Fig. 3:**
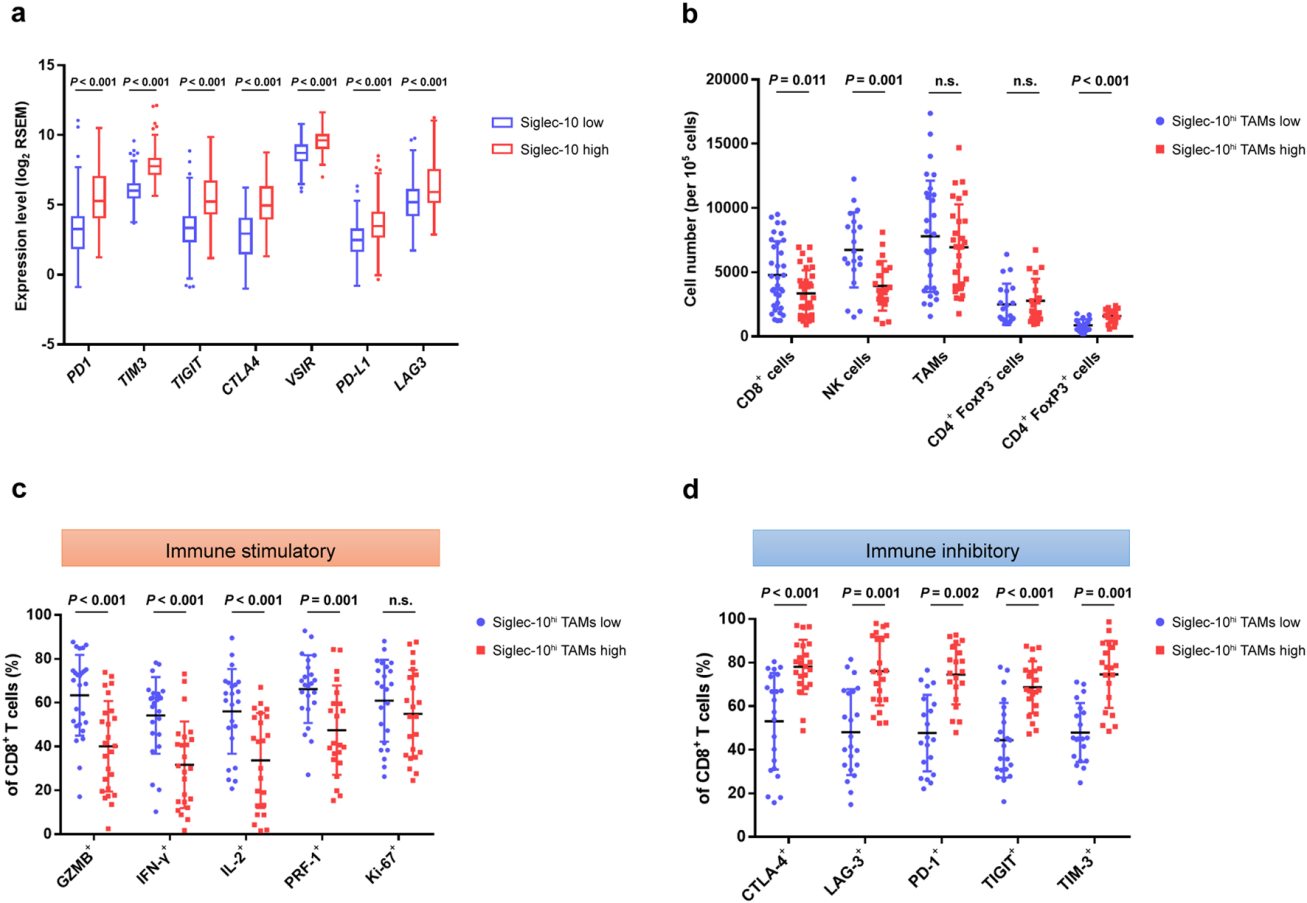
Intratumoral Siglec-10^hi^ TAMs are associated with the pro-tumor immune contexture in HCC. **a** Comparison of inhibitory receptor gene expression level between Siglec-10 low and high group in TCGA cohort. **b** The number of CD8^+^, CD56^+^, CD68^+^, CD4^+^ FoxP^+^, and CD4^+^ FoxP^−^ cells in HCC specimens with low and high Siglec-10^hi^ TAMs infiltrating based on the results of flow cytometry analysis. Cells were pre-gated on CD45. **c**, **d** The ratio of effector molecule–positive CD8^+^ cells (**c**) and inhibitory receptor-positive CD8^+^ cells (**d**) in total CD8^+^ cells in HCC specimens with low and high Siglec-10^hi^ TAM infiltration

The potential impact of intratumoral Siglec-10^hi^ TAMs on HCC immune contexture was explored. According to analysis of TCGA cohort, the Siglec-10 high expressing group showed significantly higher expression of inhibitory receptor genes including *programmed cell death 1* (*PD1*), *T cell immunoglobulin and mucin domain 3* (*TIM3*), *T cell immunoreceptor with IG and ITIM domains* (*TIGIT*), *cytotoxic T-lymphocyte-associated protein 4* (*CTLA4*), *V-set immunoregulatory receptor* (*VSIR*), *CD274*, and *lymphocyte-activation gene 3* (*LAG3*) (Fig. 3a). In flow cytometry analysis, patients with high (≥ 25%) or low (< 25%) intratumoral Siglec-10^hi^ TAM infiltration (Siglec-10^hi^ CD68^+^ cells / CD68^+^ cells) were defined according to median cut-off value. HCC with high Siglec-10^hi^ TAM infiltration exhibited higher levels of CD4^+^ FoxP3^+^ regulatory T cells compared to tumors with low Siglec-10^hi^ TAM infiltration (Fig. 3b). Flow cytometry analysis confirmed that the levels of CD45^+^ CD8^+^ T cells and CD45^+^ CD56^+^ NK cells were significantly higher in specimens with low Siglec-10^hi^ TAM infiltration compared with high Siglec-10^hi^ TAM infiltrating samples (Fig. 3b).

These results indicate a close relationship between intratumoral Siglec-10^hi^ TAM infiltration and T-cell immunity in HCC. Subsequently, the associations of intratumoral Siglec-10^hi^ TAM infiltration and different subtypes of CD8^+^ cytotoxic T lymphocytes (CTLs) were investigated. HCC specimens with high Siglec-10^hi^ TAM infiltration exhibited lower proportions of granzyme B^+^ (GZMB^+^), IFN-γ^+^, IL-2^+^ and perforin-1^+^ (PRF-1^+^) CD8^+^ CTLs (Fig. 3c), but higher proportions of CTLA-4^+^, LAG-3^+^, PD-1^+^, TIGIT^+^ and TIM-3^+^ CD8^+^ CTLs (Fig. 3d) compared to samples with low Siglec-10^hi^ TAM infiltration. These results clearly indicate that Siglec-10^hi^ TAMs may suppress the anti-tumor activity of CD8^+^ CTLs.

### Intratumoral Siglec-10^hi^ TAMs exhibit mixed M1/M2 phenotype and immunosuppressive function

**Fig. 4:**
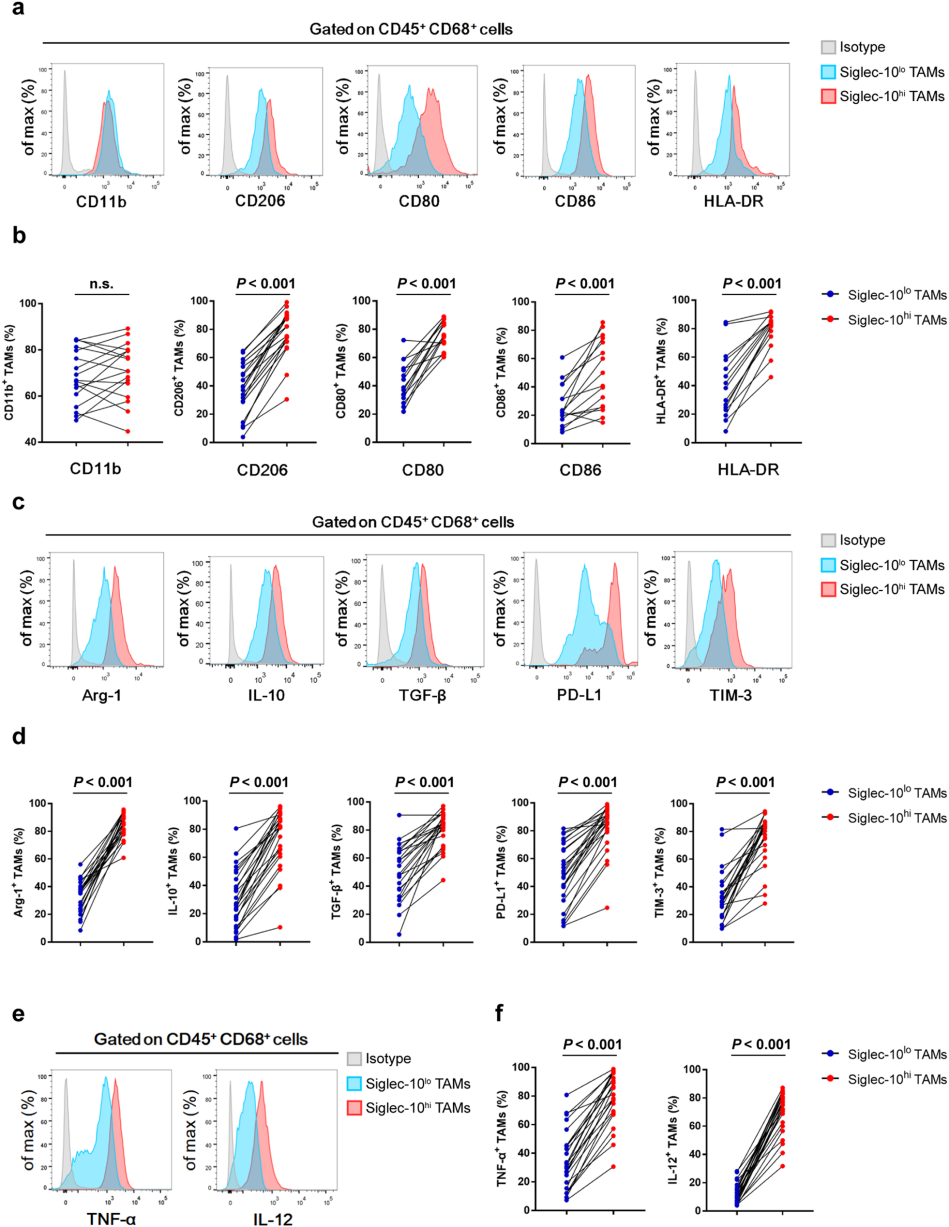
Intratumoral Siglec-10^hi^ TAMs exhibit mixed M1/M2 phenotype and immunosuppressive function. **a** Representative flow cytometry histograms showed CD11b, CD206, CD80, CD86 and HLA-DR expression in Siglec-10^hi^ and Siglec-10^lo^ TAMs in HCC tissues. TAMs were pre-gated on CD45 and CD68. **b** Statistical analysis of CD11b, CD206, CD80, CD86 and HLA-DR expression in Siglec-10^hi^ and Siglec-10^lo^ TAMs in fresh human HCC specimens. Data were analyzed with paired Student’s *t-*test. **c** Representative flow cytometry histograms showed the expression of immune inhibitory molecules in Siglec-10^hi^ and Siglec-10^lo^ TAMs. TAMs were pre-gated on CD45 and CD68. **d** Statistical analysis of immune inhibitory molecules expression in Siglec-10^hi^ and Siglec-10^lo^ TAMs. Data were analyzed with paired Student’s *t*-test. **e** Representative flow cytometry histograms showed the expression of pro-inflammatory cytokines in Siglec-10^hi^ and Siglec-10^lo^ TAMs. TAMs were pre-gated on CD45 and CD68. **f** Statistical analysis of pro-inflammatory cytokines expression in Siglec-10^hi^ and Siglec-10^lo^ TAMs. Data were analyzed using Student’s *t*-test

To explain the prognosis stratification and the difference in immune contexture, we evaluated the difference between intratumoral Siglec-10^hi^ and Siglec-10^lo^ TAMs from the same tumor. Flow cytometry analysis showed that intratumoral Siglec-10^hi^ TAMs had higher expression of both M2 macrophage marker (CD206) and M1 macrophage marker (CD80, CD86, HLA-DR) compared with Siglec-10^lo^ TAMs (Fig. 4a, b). Notably, multiple immune inhibitory markers including arginase 1 (Arg-1), interleukin 10 (IL-10), transforming growth factor beta (TGF-β), T-cell immunoglobulin and mucin domain 3 (TIM-3), and programmed death-ligand 1 (PD-L1) were strongly enriched in Siglec-10^hi^ TAMs (Fig. 4c, d). Meanwhile, intratumoral Siglec-10^hi^ TAMs also expressed higher levels of pro-inflammatory cytokines, including IL-12 and tumor necrosis factor α (TNF-α), compared to Siglec-10^lo^ TAMs (Fig. 4e, f).

**Fig. 5:**
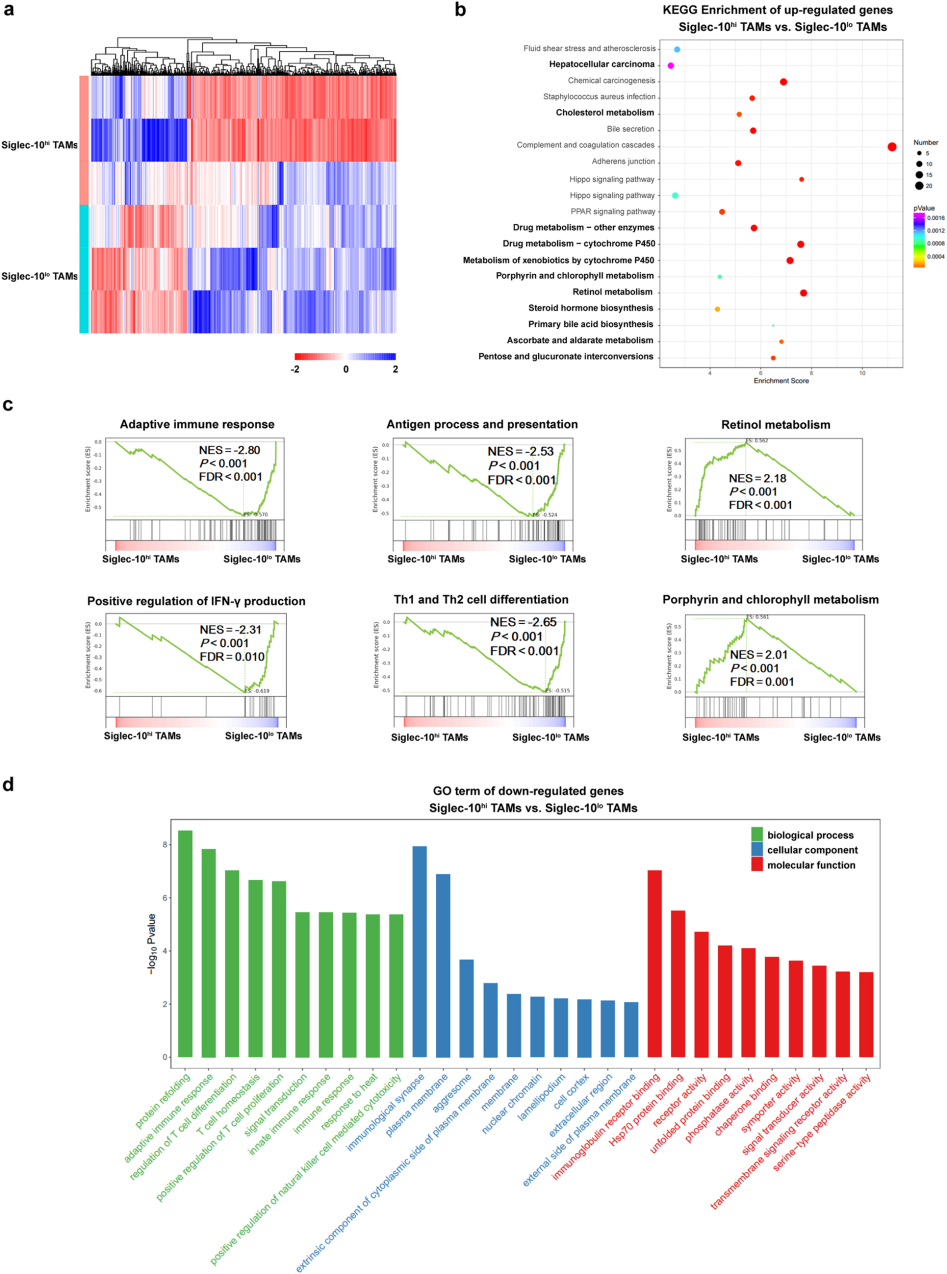
RNA-seq analysis reveals significant transcriptomic differences between Siglec-10^hi^ and Siglec-10^lo^ TAMs. **a** Heat map analysis showed gene expression differences between intratumoral Siglec-10^hi^ and Siglec-10^lo^ TAMs. **b** KEGG pathway analysis of the upregulated DEGs between intratumoral Siglec-10^hi^ and Siglec-10^lo^ TAMs. **c** GSEA plots of gene expression changes in Siglec-10^hi^ TAMs compared with Siglec-10^lo^ TAMs. The signature was defined by genes with significant expression changes. **d** GO enrichment of the downregulated DEGs between intratumoral Siglec-10^hi^ and Siglec-10^lo^ TAMs

We further investigated the transcriptomic profiling of intratumoral Siglec-10^hi^ TAMs by RNA-seq (Fig. 5a). KEGG enrichment analysis revealed that compared with Siglec-10^lo^ TAMs, intratumoral Siglec-10^hi^ TAMs exhibited an apparently enriched pathway involved with HCC and metabolic pathways (Fig. 5b). According to the results of GSEA, intratumoral Siglec-10^hi^ TAMs showed obviously downregulated genes related to adaptive immune response, antigen process and presentation, positive regulation of interferon gamma (IFN-γ) production, and T helper type 1 (Th1) and Th2 cell differentiation, whereas intratumoral Siglec-10^hi^ TAMs showed marked upregulation of genes involved in metabolic pathways (Fig. 5c). In addition, Gene Ontology (GO) analysis revealed that compared with Siglec-10^lo^ TAMs, intratumoral Siglec-10^hi^ TAMs had numerous upregulated metabolic pathways and downregulated genes related to the immune response such as adaptive immune response, regulation of T cell differentiation, T cell homeostasis, positive regulation of T cell proliferation, innate immune response, positive regulation of natural killer (NK) cell mediated cytotoxicity, immunological synapse, and immunoglobulin receptor binding (Fig. 5d). Taken together, these findings suggest that intratumoral Siglec-10^hi^ TAMs exhibit mixed M1/M2 phenotype, and exert immunosuppressive function during HCC progression.

### Blocking Siglec-10 improves the anti-tumor activity of CD8^+^ CTLs and the efficacy of the PD-1 inhibitor

**Fig. 6:**
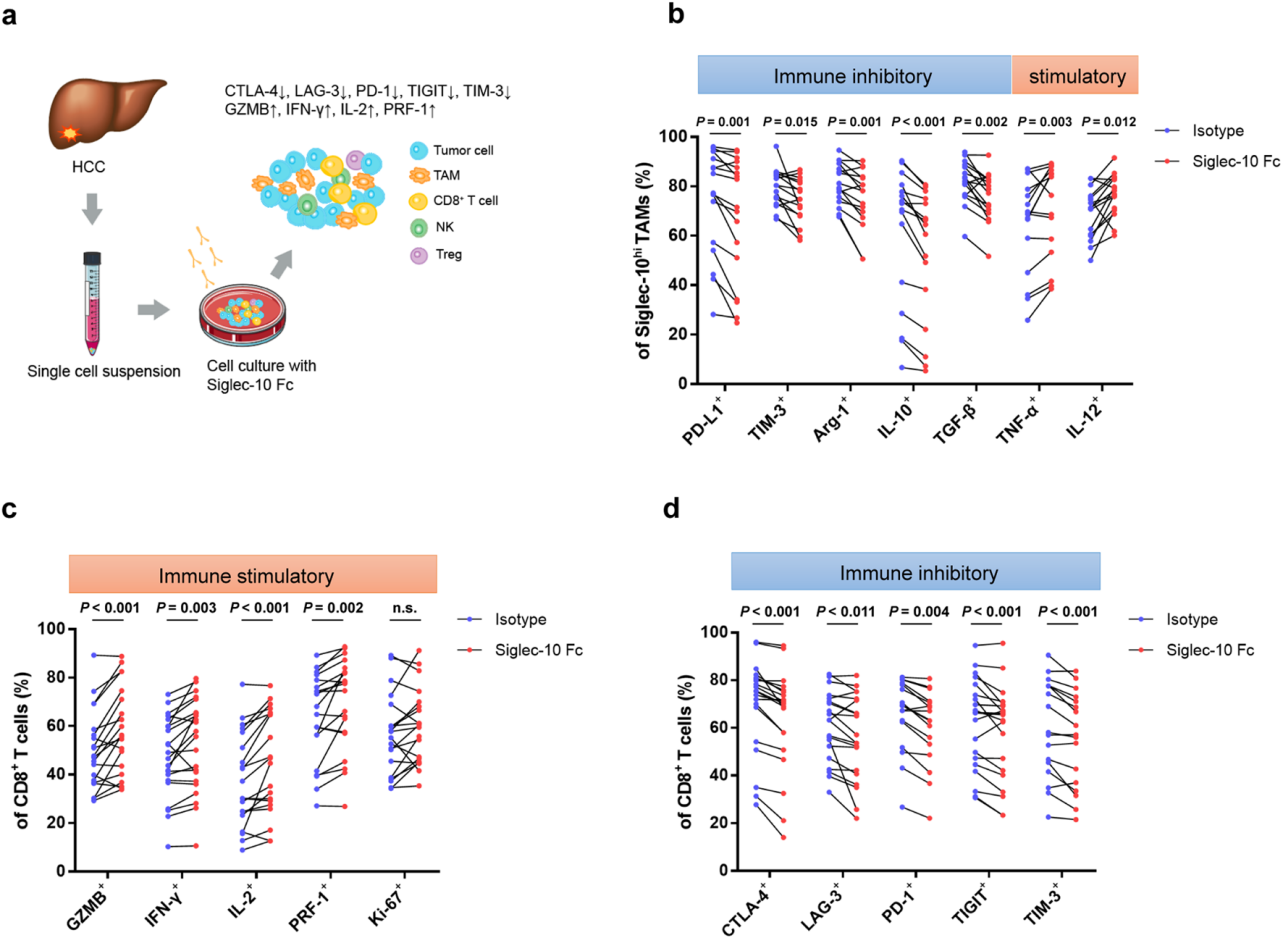
Blocking Siglec-10 restores the anti-tumor activity of CD8^+^ T cells. **a** HCC single-cell suspension was incubated with isotype control or recombinant Siglec-10 Fc chimera. **b** After incubation with isotype or Siglec-10 Fc, Siglec-10^+^ TAMs were subjected to flow cytometry analysis. **c**,** d** After incubation with isotype or Siglec-10 Fc, CD8^+^ T cells were subjected to flow cytometry analysis to detect the expression of immune stimulatory (**c**) and inhibitory (**d**) molecules

Then we analyzed the effects of blocking Siglec-10 in HCC with recombinant human Siglec-10 Fc chimera. After incubating with Siglec-10 Fc for 12 h (Fig. 6a), single-cell suspension was subjected to flow cytometry analysis to detect the phenotypes of Siglec-10^hi^ TAMs. The expression of pro-inflammatory cytokines (IL-12, TNF-α) significantly increased after blocking Siglec-10, whereas the levels of anti-inflammatory molecules were markedly decreased compared with the isotype group (Fig. 6b).

Next, the features of CD8^+^ CTLs were investigated. Compared with the isotype control, the proportion of GZMB^+^, INF-γ^+^, IL-2^+^, and PRF-1^+^ CD8^+^ CTLs was significantly increased after blocking Siglec-10 (Fig. 6c), whereas the proportion of CTLA-4^+^, LAG-3^+^, PD-1^+^, TIGIT^+^ and TIM-3^+^ CD8^+^ CTLs was markedly decreased (Fig. 6d). This suggests that blocking Siglec-10 can promote the anti-tumor activity of CD8^+^ CTLs.

**Fig. 7:**
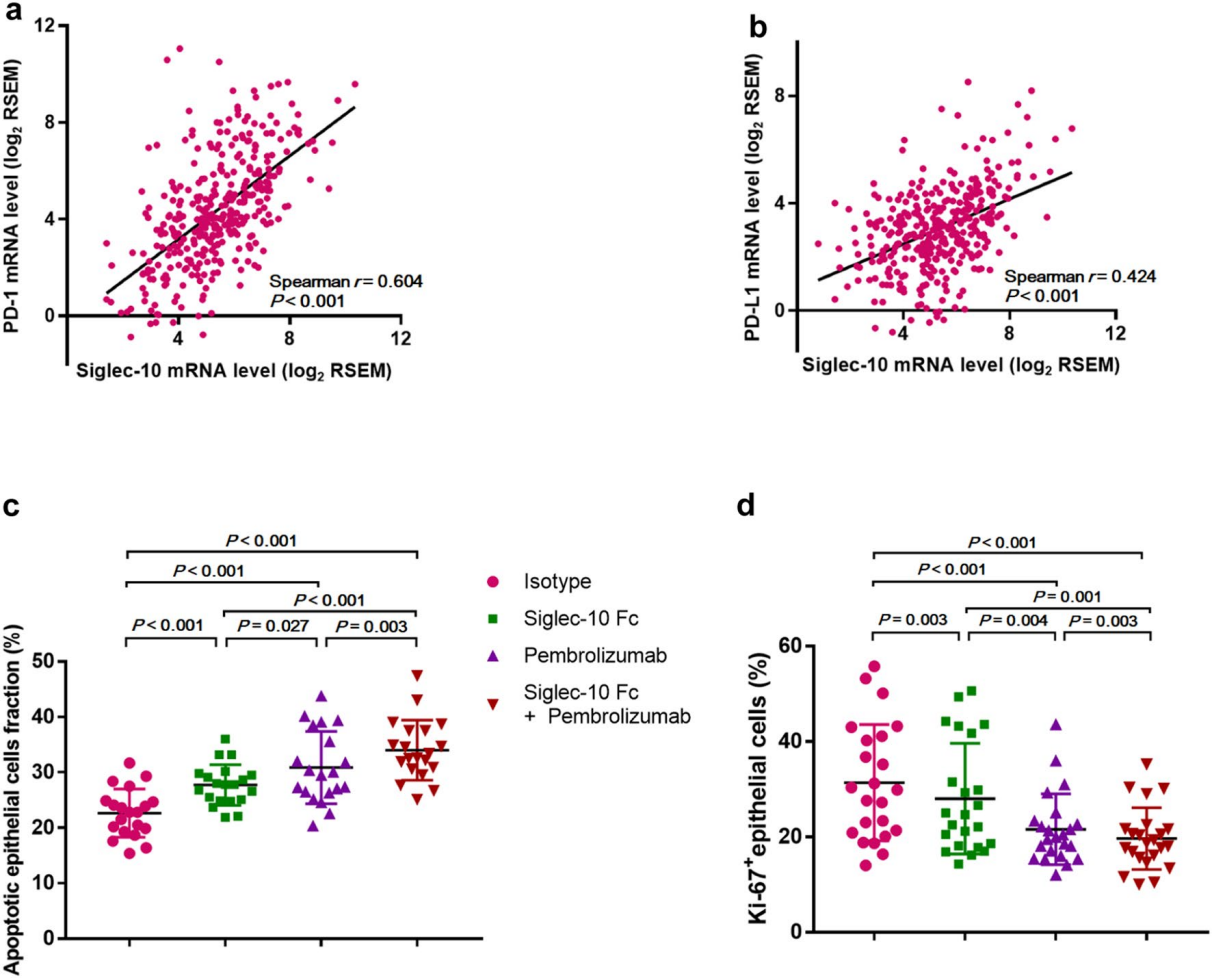
Blocking Siglec-10 produces synergistic benefits with PD-1 inhibitor**. a** Spearman correlation of the mRNA level of Siglec-10 and PD-1 in TCGA cohort. **b** Spearman correlation of the mRNA level of Siglec-10 and PD-L1 in TCGA cohort. **c** The synergistic effect of Siglec-10 Fc and pembrolizumab on the proportion of Annexin V^+^ PI^+^ epithelial cells in HCC samples (*n* = 20). **d** The synergistic effect of Siglec-10 Fc and pembrolizumab on the proportion of Ki-67^+^ epithelial cells in HCC samples (*n* = 20)

Additionally, the mRNA level of Siglec-10 was positively correlated with that of PD-1 and PD-L1 in TCGA cohort (Fig. 7a, b), indicating that Siglec-10 is involved in the PD-1 inhibitory pathway and may induce CD8^+^ CTL inactivation. Thus, we speculate that inhibiting Siglec-10 may produce synergistic benefits with the PD-1 inhibitor pembrolizumab, facilitating CD8^+^ CTL activation and tumor cell death.

We found that treatment of Siglec-10 Fc or pembrolizumab in monotherapy groups can promote CD45^−^ Epcam^+^ tumor cell death and inhibit the proliferation of tumor cells compared to the isotype control (Fig. 7c, d). Furthermore, the combination of Siglec-10 Fc and pembrolizumab had synergistic effects on promoting tumor cell apoptosis (Fig. 7c) and impairing tumor cell proliferation (Fig. 7d) compared to monotherapy groups. Overall, these findings indicate that blocking Siglec-10 may exert synergistic effects with pembrolizumab to restore the anti-tumor activity of CD8^+^ CTLs and promote tumor cell death.

## Discussion

Siglecs are proteins that bind to sugar molecules, which are attached to many other proteins. Most Siglecs act as inhibitory receptors on innate and adaptive immune cells [29]. Cancer-associated aberrant sialoglycan expression causes immune evasion upon binding to inhibitory Siglecs [30–32]. In fact, there is an association between levels of tumor glycosylation and metastatic potential [33, 34]. Notably, the high expression of Siglec-10 is associated with poor survival and immune dysfunction in patients with HCC [35]. Deficiency of Siglec-10 or blocking Siglec-10 on human macrophages increases phagocytosis and tumor cell clearance in breast cancer [14]. According to our data, intratumoral Siglec-10 was primarily co-localized with the macrophage marker CD68 in HCC tissues. This suggests that intratumoral Siglec-10^hi^ macrophages may act as an immune modulator in the TME of HCC. Indeed, our results revealed that intratumoral Siglec-10^hi^ TAMs expressed higher levels of suppressive cytokines and inhibitory receptors than intratumoral Siglec-10^lo^ TAMs. In line with these results, RNA-seq data confirmed that multiple signaling pathways involved in macrophage M2 polarization were specifically activated in Siglec-10^hi^ TAMs. Additionally, high levels of Siglec-10^hi^ TAMs infiltration were related to an immunosuppressive TME with restrained anti-tumor activity.

Sialoglycan-Siglec immune checkpoints are attractive targets for anti-tumor immunotherapy, as aberrant glycosylation is a key feature of malignant transformation [32]. The use of blocking antibodies against Siglec-7 and Siglec-9 has been successfully tested in vitro to enhance the immune response against cancer [36–38]. Siglec-2 (CD22) and Siglec-3 (CD33)-targeting antibody–drug conjugates have been approved by US Food and Drug Administration for the treatment of lymphoma and leukemia [39–41]. Previous in vivo research has reported that sialic acid blockade enhances CTL-mediated killing of tumor cells, thus suppressing tumor growth [42]. A recent study revealed that Siglec-15 suppresses antigen-specific T-cell responses in vitro and in vivo. Blocking Siglec-15 enhances anti-tumor immunity and inhibits tumor growth in mice [43]. A humanized anti-Siglec-15 monoclonal antibody is being assessed in a phase I clinical trial in advanced solid tumors (NCT03665285). Blockade of PD-1/PD-L1 can reactivate the anti-tumor function of intratumoral T cells in HCC and has shown clinical efficacy in 20% HCC patients [5]. Importantly, combining blockade of PD-L1 with TIM3, LAG3, or CTLA4 further improved the responsiveness and was effective in more patients [44]. Recent study on microenvironment characterization indicated that the immune status of HCC could help to recognize patients with different prognosis and responses to immunotherapy in HCC [45]. Suppressive immune cells inhibit the immune response of tumor-specific T cells and could serve as biomarkers of anti-PD-1 therapy [46]. In accordance with these results, we found that blocking Siglec-10 led to reduced expression of immune inhibitory molecules in the TME and enhanced the anti-tumor activity of CTLs. In addition, compared with monotherapy groups, the combination of Siglec-10 blockade and pembrolizumab significantly enhanced tumor cell apoptosis and impaired tumor cell proliferation.

Diversity and plasticity are hallmarks of the monocyte-macrophage lineage. Macrophages associated with different TME exhibit a variety of phenotypes. While the description of M1 and M2 phenotypes suggests that TAMs can be either tumor-killing or tumor-promoting, the heterogeneity of TAM functions in the TME goes far beyond that. Transcriptome analysis has revealed that macrophages have an entirely different transcriptional profile distinct from M1 or M2 activation [47–50]. TAM subtypes generally perform mixed M1/M2 markers at different levels [48, 50, 51]. In a reassessment, Fernando [52] postulated that macrophages do not form settled subsets but constantly respond to a combination of cues present in the TME.

In our study, we also found that intratumoral Siglec-10^hi^ TAMs exhibited mixed M1 (CD80, CD86, HLA-DR) and M2 (CD206) markers. Compared to Siglec-10^lo^ TAMs, Siglec-10^hi^ TAMs showed higher levels of immune stimulatory cytokines (TNF-α, IL-12), and higher levels of immune inhibitory molecules (PD-L1, TIM-3, Arg-1, IL-10, TGF-β). Interestingly, the expression of immune inhibitory molecules was significantly suppressed after blocking Siglec-10, whereas the levels of TNF-α and IL-12 were further increased. It is known that despite functional impairment, exhausted T cells with overexpression of inhibitory receptors retain a crucial level of control over tumor growth [53–55]. Targeting these inhibitory receptors could enhance T cell-mediated immune responses against tumors [55]. Likewise, we speculate that TAM subtype with M2 features, such as Siglec-10^hi^ TAMs, retain the anti-tumor effect in some degree. Indeed, our findings indicate that Siglec-10 expression may facilitate the secretion of macrophage-derived anti-inflammatory cytokines in HCC. However, once released from inhibition, Siglec-10^hi^ TAMs may be transformed into an active role in anti-tumor immunity. In summary, Siglec-10 represents a novel immune inhibitor in HCC. Targeting Siglec-10 may serve as a promising approach to restore anti-tumor immunity.

This study had several limitations. First, Siglec-10 is widely expressed on various leucocytes and predominantly distributed on macrophages in HCC tissues. In this study, we did not investigate the effects of Siglec-10^hi^ NK cells, Siglec-10^hi^ dendritic cells, or other cells expressing Siglec-10 in the TME. Second, the study lacked in vivo experiments to further characterize the role of Siglec-10^hi^ TAMs in HCC. Based on the current findings, we will further explore the mechanism underlying the results that blocking Siglec-10 promotes tumor cell death.

## Conclusion

As immune suppressors, Siglec-10^hi^ TAMs may play a key role in tumor immune evasion via expression of immune inhibitory molecules and inactivation of CD8^+^ CTLs. Blocking Siglec-10 led to markedly declined secretion of anti-inflammatory cytokines and increased the cytotoxic effects of CD8^+^ T cells against tumor cells. These findings highlight the importance of Siglec-10^hi^ TAMs in immune modulation of the TME and provide a novel promising immunotherapy approach for HCC.

## Supplementary Information

**Additional file 1: Figure S1.** Flow chart of patient selection. **Figure S2.** Gating strategy for selection of Siglec-10^hi^ macrophages. **Figure S3.** Representative images of CD8^+^ T cells by flow cytometry analysis. **Table S1.** Characteristics of HCC patients and relationship with intratumoral Siglec-10^+^ cell infiltration. **Table S2.** Immunohistochemistry, immunofluorescence and flow cytometry antibodies.

## Data Availability

The Cancer Genome Atlas Liver Hepatocellular Carcinoma (TCGA-LIHC PanCancer Atlas) mRNA and clinical data, including RNA sequencing and clinicopathological data were downloaded from https://www.cbioportal.org. Other (anonymized) data are available from the corresponding authors upon reasonable request.

## Abbreviations

TAMs: Tumor-associated macrophages
TME: Tumor microenvironment
HCC: Hepatocellular carcinoma
Siglec-10: Sialic acid-binding immunoglobulin-like lectin 10
PD-1: Programmed cell death protein 1
OS: Overall survival
RFS: Recurrence-free survival
RNA-seq: RNA sequencing
HR: Hazard ratio
CI: Confidence interval
