# Supplementary material for: Blocking siglec-10hi tumor-associated macrophages improves anti-tumor immunity and enhances immunotherapy for hepatocellular carcinoma
Source: Exp Hematol Oncol. 2021 Jun 10;10:36. doi: 10.1186/s40164-021-00230-5 (PMC8191104; doi:10.1186/s40164-021-00230-5)
Supplement: Supplementary file 1 — Additional file 1: Figure S1. Flow chart of patient selection. Figure S2. Gating strategy for selection of Siglec-10hi macrophages. Figure S3. Representative images of CD8+ T cells by flow cytometry analysis. Table S1. Characteristics of HCC patients and relationship with intratumoral Siglec-10+ cell infiltration. Table S2. Immunohistochemistry, immunofluorescence and flow cytometry antibodies. [file 40164_2021_230_MOESM1_ESM.doc]

**SUPPLEMENTARY MATERIAL**

**Contents**

**Supplementary Figure S1. Flow chart of patient selection.**

**Supplementary Figure S2. Gating strategy for selection of Siglec-10^hi^ macrophages.**

**Supplementary Figure S3. Representative images of CD8^+^ T cells by flow cytometry analysis.**

**Supplementary Table S1. Characteristics of HCC patients and relationship with intratumoral Siglec-10^+^ cell infiltration.**

**Supplementary Table S2. Immunohistochemistry, immunofluorescence and flow cytometry antibodies.**

**Supplementary Figure S1.**


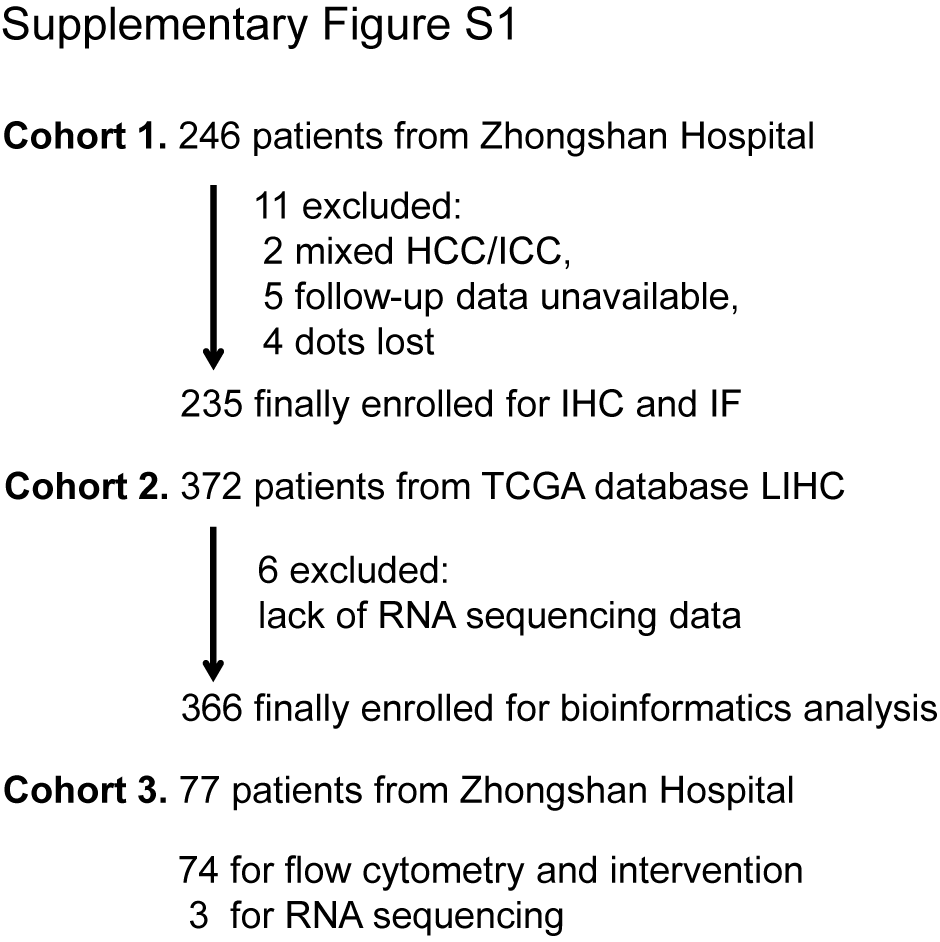


**Supplementary Figure S1. Flow chart of patient selection.** The inclusion and exclusion process of the cohorts.

**Supplementary Figure S2**

­
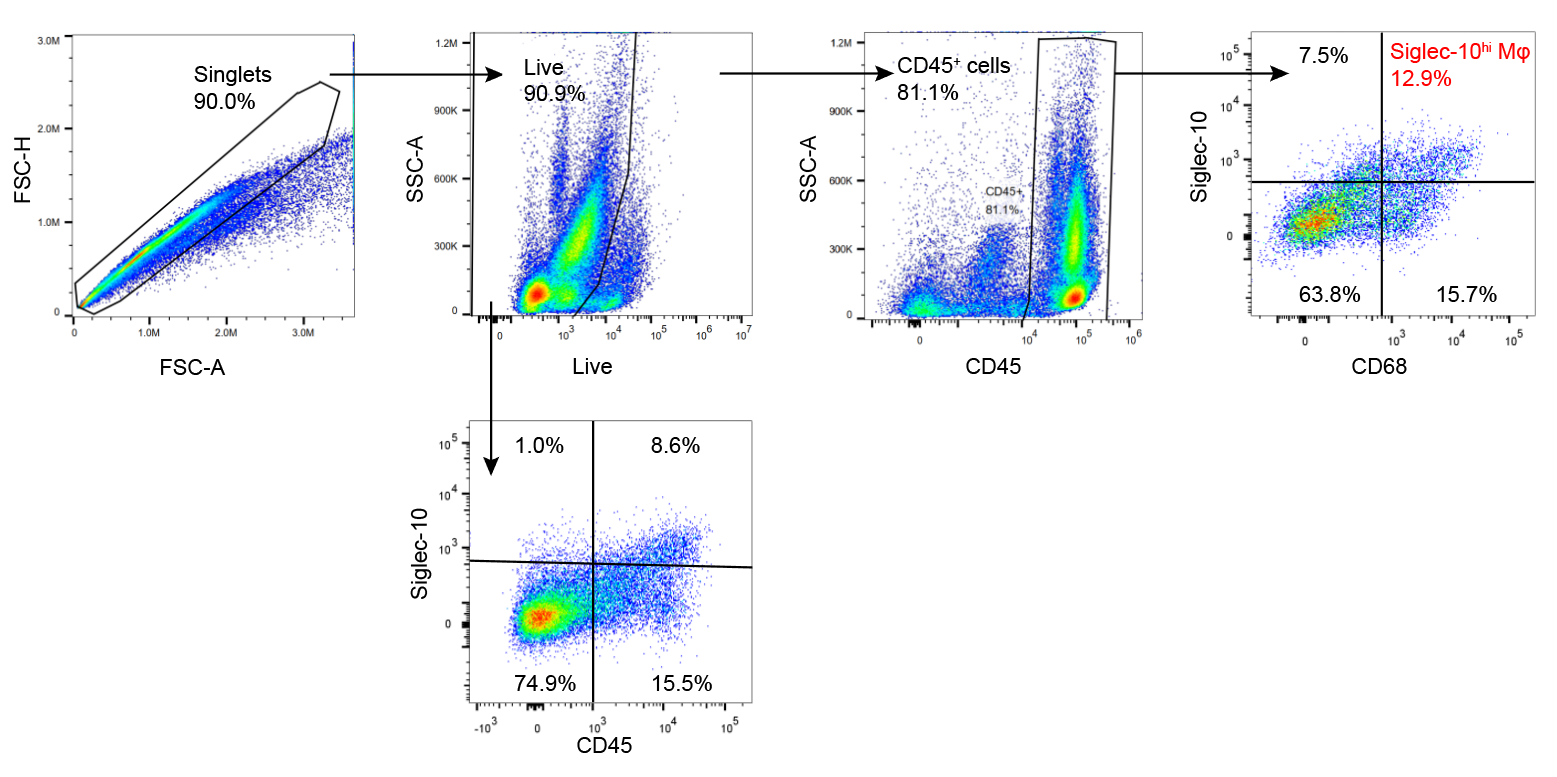


**Supplementary Figure S2. Gating strategy for selection of Siglec-10^hi^ macrophages.** Gating strategy for selection of Siglec-10^hi^ CD68^+^ macrophages. Arrows indicate directionality of sub-gates. Identified populations are marked in red font.

**Supplementary Figure S3**
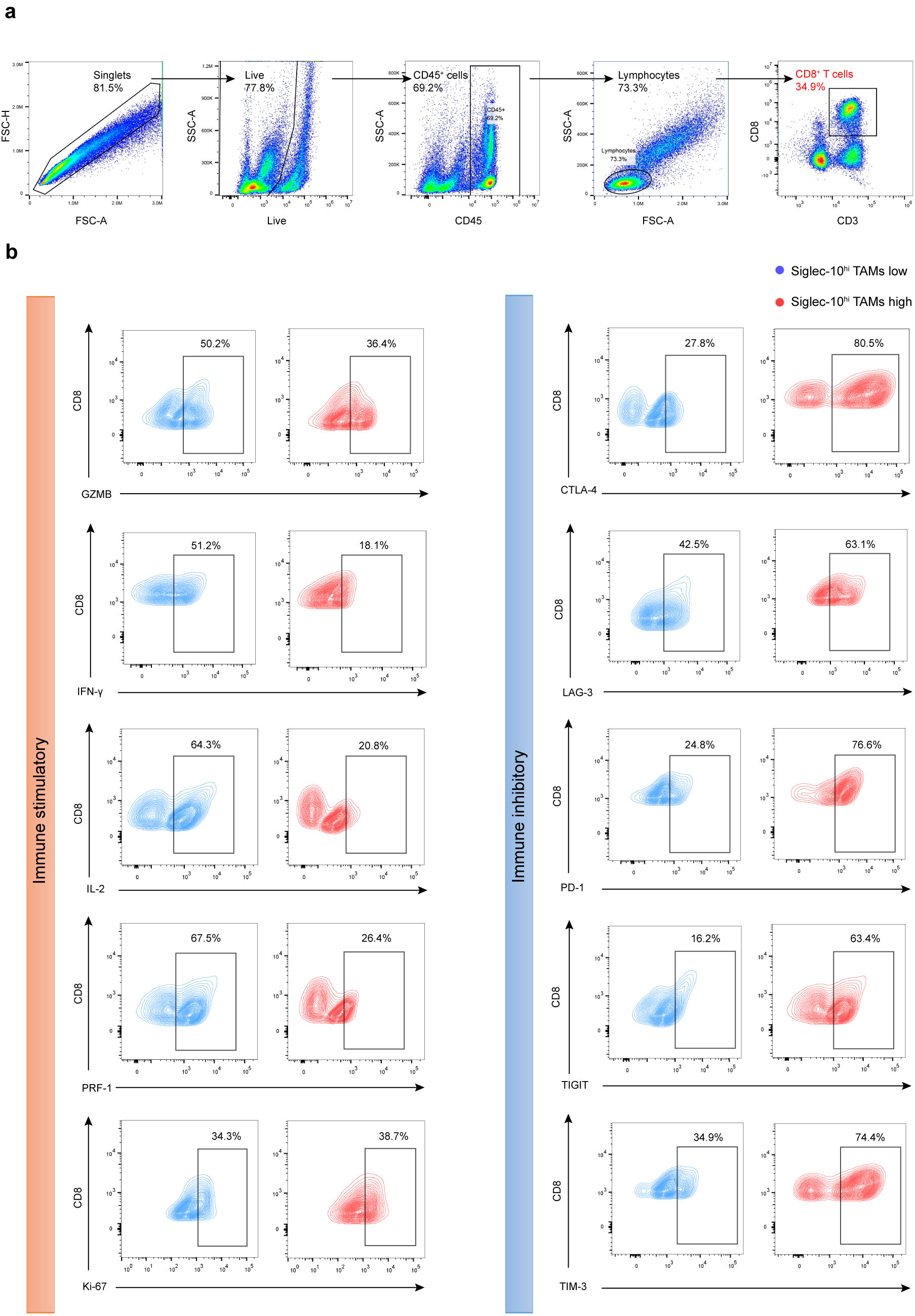


**Supplementary Figure S3. Representative images of CD8^+^ T cells by flow cytometry analysis.** **a** Gating strategy for selection of CD8^+^ T cells. Arrows indicate directionality of sub-gates. Identified populations are marked in red font. **b** Representative images of CD8^+^ T cells in CD45^+^ cells. The expression of immune stimulatory molecules (including GZMB, IFN-γ, IL-2, PRF-1 and Ki-67), and immune inhibitory receptors (including PD-1, LAG-3, PD-1, TIGIT and TIM-3) in CD8^+^ T cells between low and high Siglec-10^hi^ TAMs infiltration.

**Supplementary Table S1.** Characteristics of HCC patients and relationship with intratumoral Siglec-10^+^ cell infiltration

| | **Characteristics**  **(Number of patients %)** | **Discovery set (*n* = 115)** | | |  | **Validation set (*n* = 120)** | | | | --- | --- | --- | --- | --- | --- | --- | --- | | **High (*n* = 51)** | **Low (*n* = 64)** | ***P*** |  | **High (*n* = 52)** | **Low (*n* = 68)** | ***P*** | | **Age (y)** |  |  | 0.818 |  | |  | 0.296 | | **≤50** | 21 (41) | 25 (39) |  | 13 (25) | | 23 (34) |  | | **>50** | 30 (59) | 39 (61) |  | 39 (75) | | 45 (66) |  | | **Gender** |  |  | 0.677 |  | |  | 0.781 | | **Male** | 45 (88) | 58 (91) |  | 45 (87) | | 60 (89) |  | | **Female** | 6 (11) | 6 (9) |  | 7 (13) | | 8 (11) |  | | **Cirrhosis** |  |  | 0.437 |  | |  | 0.142 | | **Absent** | 21 (41) | 31 (48) |  | 22 (42) | | 20 (29) |  | | **Present** | 30 (58) | 33 (52) |  | 30 (58) | | 48 (71) |  | | **HBsAg** |  |  | 0.679 |  | |  | 0.534 | | **Negative** | 13 (25) | 19 (30) |  | 15 (29) | | 16 (24) |  | | **Positive** | 38 (75) | 45 (70) |  | 37 (71) | | 52 (76) |  | | **Child-Pugh score** |  |  | 0.650 |  | |  | 0.231 | | **A** | 44 (86) | 57 (89) |  | 39 (75) | | 57 (84) |  | | **B** | 7 (14) | 7 (11) |  | 13 (25) | | 11 (16) |  | | **ALT (U/L)** |  |  | 0.372 |  | |  | 0.602 | | **≤50** | 34 (67) | 37 (58) |  | 36 (69) | | 44 (65) |  | | **>50** | 17 (33) | 27 (42) |  | 16 (31) | | 24 (35) |  | | **AFP (ng/ml)** |  |  | **0.011** |  | |  | **0.027** | | **≤20** | 15 (29) | 34 (53) |  | 17 (33) | | 36 (53) |  | | **>20** | 36 (71) | 30 (47) |  | 35 (67) | | 32 (47) |  | | **Tumor size (cm)** |  |  | 0.248 |  | |  | 0.271 | | **≤5** | 31 (61) | 32 (50) |  | 20 (38) | | 33 (49) |  | | **>5** | 20 (39) | 32 (50) |  | 32 (62) | | 35 (51) |  | | **Tumor multiplicity** |  |  | 0.675 |  | |  | 0.196 | | **Solitary** | 34 (67) | 45 (70) |  | 30 (58) | | 47 (69) |  | | **Multiple** | 17 (33) | 19 (30) |  | 22 (42) | | 21 (31) |  | | **Vascular invasion** |  |  | **0.042** |  | |  | **0.038** | | **Absent** | 23 (45) | 41 (64) |  | 23 (44) | | 43 (63) |  | | **Present** | 28 (55) | 23 (36) |  | 29 (58) | | 25 (37) |  | | **Fibrous capsule** |  |  | 0.722 |  | |  | 0.554 | | **Absent** | 28 (55) | 33 (52) |  | 25 (48) | | 29 (43) |  | | **Present** | 23 (45) | 31 (48) |  | 27 (52) | | 39 (57) |  | | **TNM stage** |  |  | 0.071 |  | |  | 0.373 | | **I** | 12 (24) | 23 (36) |  | 17 (33) | | 20 (30) |  | | **II** | 36 (71) | 32 (50) |  | 31 (60) | | 39 (57) |  | | **III** | 3 (5) | 9 (14) |  | 14 (7) | | 9 (13) |  | |
| --- | --- | --- | --- | --- | --- | --- | --- | --- | --- | --- | --- | --- | --- | --- | --- | --- | --- | --- | --- | --- | --- | --- | --- | --- | --- | --- | --- | --- | --- | --- | --- | --- | --- | --- | --- | --- | --- | --- | --- | --- | --- | --- | --- | --- | --- | --- | --- | --- | --- | --- | --- | --- | --- | --- | --- | --- | --- | --- | --- | --- | --- | --- | --- | --- | --- | --- | --- | --- | --- | --- | --- | --- | --- | --- | --- | --- | --- | --- | --- | --- | --- | --- | --- | --- | --- | --- | --- | --- | --- | --- | --- | --- | --- | --- | --- | --- | --- | --- | --- | --- | --- | --- | --- | --- | --- | --- | --- | --- | --- | --- | --- | --- | --- | --- | --- | --- | --- | --- | --- | --- | --- | --- | --- | --- | --- | --- | --- | --- | --- | --- | --- | --- | --- | --- | --- | --- | --- | --- | --- | --- | --- | --- | --- | --- | --- | --- | --- | --- | --- | --- | --- | --- | --- | --- | --- | --- | --- | --- | --- | --- | --- | --- | --- | --- | --- | --- | --- | --- | --- | --- | --- | --- | --- | --- | --- | --- | --- | --- | --- | --- | --- | --- | --- | --- | --- | --- | --- | --- | --- | --- | --- | --- | --- | --- | --- | --- | --- | --- | --- | --- | --- | --- | --- | --- | --- | --- | --- | --- | --- | --- | --- | --- | --- | --- | --- | --- | --- | --- | --- | --- | --- | --- | --- | --- | --- | --- | --- | --- | --- | --- | --- | --- | --- | --- | --- | --- | --- | --- | --- | --- | --- | --- | --- | --- | --- | --- | --- | --- | --- | --- | --- | --- | --- | --- | --- | --- | --- | --- | --- | --- | --- | --- | --- | --- | --- | --- | --- | --- | --- | --- | --- | --- | --- | --- | --- | --- | --- | --- | --- | --- | --- | --- | --- | --- | --- | --- | --- | --- | --- | --- | --- | --- | --- | --- | --- | --- | --- | --- | --- | --- | --- | --- | --- | --- | --- | --- | --- | --- | --- | --- | --- |

**Supplementary Table S2.** Immunohistochemistry, immunofluorescence and flow cytometry antibodies

| **Markers** | **Antibody names** | **Provider** | **Host** | **Catalog No.** | **Concentration** |
| --- | --- | --- | --- | --- | --- |
| Siglec-10 | Anti-Human SIGLEC10 Antibody | Abcam | Rb | ab198724 | 1:200 |
| CD68 | Anti-Human CD68 Antibody | Abcam | Ms | ab955 | 1:400 |
| CD45 | APC-Cy7 Anti-Human CD45 Antibody | Biolegend | Ms | 368516 | 5 ul / test |
| CD45 | PE-Cy7 Anti-Human CD45 Antibody | Biolegend | Ms | 368532 | 5 ul / test |
| CD14 | FITC Anti-Human CD14 Antibody | BD | Ms | 555397 | 20 ul / test |
| CD3 | PE-Cy7 anti-human CD3 Antibody | Biolegend | Ms | 100220 | 5 ul / test |
| CD4 | Brilliant Violet® 421 Anti-Human CD4 Antibody | BD | Ms | 562424 | 5 ul / test |
| CD8 | BB515 Anti-Human CD8 Antibody | BD | Ms | 564526 | 5 ul / test |
| CD19 | PerCP/Cy5.5 anti-human CD19 Antibody | Biolegend | Ms | 363016 | 5 ul / test |
| CD56 | FITC anti-human CD56 Antibody | Biolegend | Ms | 362546 | 5 ul / test |
| CD11c | Brilliant Violet® 605 Anti-Human IL-2 Antibody | Biolegend | Ms | 301636 | 5 ul / test |
| CD326 | CD326 (Epcam) Monoclone Antibody, eFluor660 | eBioscience | Ms | 50-9326-42 | 5 ul / test |
| CD206 | FITC Anti-Human CD206 Antibody | BD | Ms | 551135 | 20 ul / test |
| CD11b | FITC anti-human CD11b Antibody | Biolegend | Ms | 301330 | 5 ul / test |
| CD80 | PE-Cy7 Anti-Human CD80 Antibody | Biolegend | Ms | 305218 | 5 ul / test |
| CD86 | Brilliant Violet® 650 Anti-Human CD86 Antibody | Biolegend | Ms | 305428 | 5 ul / test |
| HLA-DR | APC Anti-Human HLA-DR Antibody | Biolegend | Ms | 307610 | 5 ul / test |
| CD68 | Alexa Fluor® 647 Anti-Human CD68 Antibody | BD | Ms | 562111 | 5 ul / test |
| Siglec-10 | PE anti-human Siglec-10 Antibody | Biolegend | Ms | 347604 | 5 ul / test |
| IFN-γ | PE-Cy7 Anti-Human IFN-γ Antibody | BD | Ms | 557643 | 5 ul / test |
| GZMB | AF647 anti-Human Granzyme B Antibody | BD | Ms | 560212 | 5 ul / test |
| GZMB | PE Anti-Human Granzyme B Antibody | BD | Ms | 561142 | 5 ul / test |
| PRF-1 | Alexa Fluor® 647 Anti-Human Perforin Antibody | BD | Ms | 563576 | 5 ul / test |
| IL2 | Brilliant Violet® 605 Anti-Human IL-2 Antibody | Biolegend | Ms | 500332 | 5 ul / test |
| TNF-α | Alexa Fluor® 647 anti-human TNF-α Antibody | Biolegend | Ms | 502916 | 5 ul / test |
| IL12 | L-12 Monoclonal Antibody, eFluor 660, eBioscience™ | eBioscience | Ms | 50-7359-42 | 5 ul / test |
| Arg-1 | APC anti-human Arginase I Antibody | Biolegend | Ms | 369706 | 5 ul / test |
| IL10 | IL-10 Monoclonal Antibody, eFluor 660 | eBioscience | Ms | 50-7108-42 | 5 ul / test |
| TGF-β | PE/Cy7 anti-human LAP (TGF-β1) Antibody | Biolegend | Ms | 349610 | 5 ul / test |
| PD-L1 | Brilliant Violet 785™ anti-human CD274 ( PD-L1) Antibody | Biolegend | Ms | 329736 | 5 ul / test |
| TIM-3 | PE Anti-Human TIM-3 (CD366) Antibody | BD | Ms | 563422 | 5 ul / test |
| CTLA-4 | BV605 anti-human CD152 (CTLA-4) Antibody | Biolegend | Ms | 369610 | 5 ul / test |
| LAG-3 | BV785 anti-human CD223 (LAG-3) Antibody | Biolegend | Ms | 369322 | 5 ul / test |
| TIGIT | APC anti-human TIGIT Antibody | Biolegend | Ms | 372706 | 5 ul / test |
| PD-1 | AF647 anti-Human CD279 (PD-1) Antibody | BD | Ms | 560838 | 5 ul / test |
| Foxp3 | Alexa Fluor® 647 anti-Human FoxP3 Antibody | BD | Ms | 561184 | 5 ul / test |
| Ki67 | AF700 anti-human Ki-67 Antibody | Biolegend | Ms | 350530 | 5 ul / test |
